# Effect of PGC1-beta ablation on myonuclear organisation

**DOI:** 10.1007/s10974-019-09549-3

**Published:** 2019-09-05

**Authors:** Ryan Beedour, Jacob A. Ross, Yotam Levy, Julien Ochala

**Affiliations:** Faculty of Life Sciences & Medicine, School of Basic and Medical Biosciences, Centre of Human and Applied Physiological Sciences, King’s College London, London, UK

**Keywords:** PGC1-beta, Myonuclear domain, Myofibre, Nuclear shape

## Abstract

Skeletal muscle fibres are large, elongated multinucleated cells. Each nucleus within a myofibre is responsible for generating gene products for a finite volume of cytoplasm—the myonuclear domain (MND). Variation in MND sizes during atrophy, hypertrophy and disease states, are common. The factors that contribute to definitive MND sizes are not yet fully understood. Previous work has shown that peroxisome proliferator-activated receptor gamma coactivator 1α (PGC1-α) modulates MND volume, presumably to support increased biogenesis of mitochondria. The transcriptional co-regulator peroxisome proliferator-activated receptor gamma coactivator 1β (PGC1-β) is a homologue of PGC1-α with overlapping functions. To investigate the role of this protein in MND size regulation, we studied a mouse skeletal muscle specific knockout (cKO). Myofibres were isolated from the fast twitch extensor digitorum longus (EDL) muscle, membrane-permeabilised and analysed in 3 dimensions using confocal microscopy. PGC1-β ablation resulted in no significant difference in MND size between cKO and wild type (WT) mice, however, subtle differences in nuclear morphology were observed. To determine whether these nuclear shape changes were associated with alterations in global transcriptional activity, acetyl histone H3 immunostaining was carried out. We found there was no significant difference in nuclear fluorescence intensity between the two genotypes. Overall, the results suggest that PGC-1α and PGC-1β play different roles in regulating nuclear organisation in skeletal muscle; however, further work is required to pinpoint their exact functions.

## Introduction

A skeletal muscle fibre (also known as a myofibre) is typically 10 to 80 µm in diameter and can be up to 25 cm in length. Myofibres of this size cannot be supported by a single myonucleus (Hall and Ralston 1989; Edgerton and Roy 1991; Ralston and Hall 1992). Hundreds of nuclei are then needed to support and coordinate the large and lengthy myofibres (Hall and Ralston 1989; Edgerton and Roy 1991; Ralston and Hall 1992; Levy et al. 2018). Along with osteoclasts and cytotrophoblasts, skeletal muscle fibres are one of only three truly multinucleated cell types in the body. Surrounding each nucleus is a theoretical territory of cytoplasm for which gene expression is controlled—the myonuclear domain (MND) (Hall and Ralston 1989; Edgerton and Roy 1991; Ralston and Hall 1992; Levy et al. 2018). Nuclei help the generation of proteins for their respective MNDs to meet the changing demands of the cells. Theoretically, if MNDs are too large, portions of the fibre may not receive enough mRNA or proteins necessary for function, leading to dysfunction (Qaisar et al. 2012; Levy et al. 2018). Undersized MNDs may lead to overlapping regions that cause excessive stress on each myonuclei, again resulting in dysfunction. Hence, it is imperative that nuclear number and MNDs remain optimal. However, despite its crucial importance, the signalling pathways fine tuning MND volumes remain unclear.

**Fig. 1:**
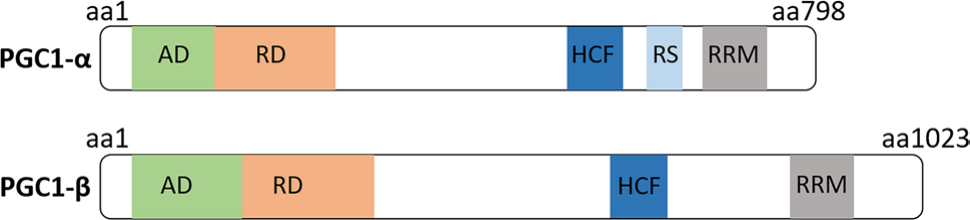
Structural and functional domains of PGC1- α and PGC1-β. The N-terminal region contains (i) a highly conserved activation domain (AD); as well as (ii) a region responsible for the inhibition of PGC1-α and PGC1-β activity (RD). The C-terminus has (i) a HCF binding site, which is implicated in cell cycle regulation; and (ii) multiple domains involved in mRNA splicing such as RRM and RS (only for PGC1-α)

We have recently demonstrated that the transcriptional coactivator, peroxisome proliferator-activated receptor-γ coactivator 1-α (PGC-1α), plays an essential role in muscle metabolism in general, and the determination of MND sizes in particular (Ross et al. 2017). When PGC1-α is overexpressed, MNDs get smaller to cope with the accrued mitochondrial biogenesis and increased demand for bioenergetic proteins per se (Ross et al. 2017). PGC1-β is a homologue of PGC1-α, with some overlapping structures (Fig. 1) and functions (Gali Ramamoorthy et al. 2015). Indeed, they both modulate mitochondrial biogenesis with few subtle differences (Gali Ramamoorthy et al. 2015). PGC1-α regulates the adaptation to exercise whilst PGC1-β only intervenes in the basal metabolic regulation. Based on the similarities between these two isoforms, in the present study we aimed to test the hypothesis that modifying PGC1-β content would impact myonuclear number and MNDs. For this, we used mice with a skeletal muscle-specific ablation of PGC1-β. We then isolated myofibres and run a 3D analysis of myonuclear organisation (Ross et al. 2017, 2018).

## Materials and methods

### Animals

The generation of the mice involved tamoxifen-dependent Cre-ER^T2^ recombinase under the control of the HSA regulatory elements (Gali Ramamoorthy et al. 2015). PGC1-β^(i)skm-/-^ (PGC-1β cKO) mice and control littermates were as described previously (Gali Ramamoorthy et al. 2015). Mice were maintained in a temperature and humidity controlled animal facility, with a 12-h light/dark cycle. Standard rodent chow 2800 kcal kg^−1^ (Usine d’Alimentation Rationelle, Villemoisson-sur-Orge, France) and water was provided ad libitum. Breeding and maintenance of mice were performed in the accredited IGBMC/ICS animal house (A67-218-37 notification of 16/10/2013), in compliance with French and EU regulations on the use of laboratory animals for research, under the supervision of DM who holds animal experimentation authorizations from the French Ministry of agriculture and Fisheries (N 67-209 and A 67-227). Animals were killed by cervical dislocation.

### Solutions

Relaxing solution contained 4 mM Mg-ATP, 1 mM free Mg^2+^, 20 mM imidazole, 7 mM EGTA, 14.5 mM creatine phosphate, and KCl to adjust the ionic strength to 180 mM and pH to 7.0. The concentration of free Ca^2+^ was 10^−9^ M.

### Myofibre permeabilisation

Right after dissection, the tibialis anterior was treated with skinning solution (relaxing solution containing glycerol; 50:50 v/v) for 24 h at 4 °C, after which they were transferred to − 20 °C (Frontera and Larsson 1997).

### Myonuclear organisation of single myofibres

On the day of experiment (within 4 weeks of muscle dissection), single myofibres were randomly isolated. Arrays of approximately nine myofibres were prepared at room temperature (RT). For each myofibre, both ends were clamped to half-split copper meshes designed for electron microscopy (SPI G100 2010C-XA, width, 3 mm), which had been glued to cover slips (Menzel-Gläser, 22 × 50 mm, thickness 0.13–0.16 mm). For the measurement of nuclear coordinates, fibres were mounted at a fixed sarcomere length of ≈ 2.20 µm. This was a prerequisite for exact determination of nuclei spatial organisation as it allowed accurate comparisons between myofibres (Ross et al. 2017, 2018). Note that our procedure of membrane-permeabilisation is well-known to remove all the satellite cells at the periphery of muscle fibres, hence, what remains is myonuclei only.

Arrays were subsequently fixed in 4% PFA/PBS, and further permeabilised with 0.1% Triton-X100/PBS for 10 min each. Fibres were then subjected to actin staining (Rhodamine-conjugated Phalloidin, Molecular Probes, R415, 1:100 in PBS) and nuclear staining (DAPI, Molecular Probes, D3571, 1:1000 in PBS). Images were acquired using a confocal microscope (Zeiss Axiovert 200, objective × 20) equipped with CARV II confocal imager (BD Bioscience). To visualise muscle fibres in 3D, stacks of 100 images were acquired (1 µm *z* increments) and analysed with a custom-made Matlab programme. A distribution score (‘g’) is a measure of the regularity of spacing between nuclei; this was calculated as described before (Bruusgaard et al. 2003). Briefly, a theoretical optimum distribution (M_O_) and a theoretical random distribution (M_R_) were generated for each fibre, based on the numbers and coordinates of the corresponding myonuclei. This was compared with the experimental distribution (M_E_), and a ‘g’ score was calculated with the equation: g = (M_E _− M_R_)/(M_O _− M_R_).

Acetyl-Histone H3 (Lys9/Lys14) was visualised using the following primary antibody: Cell Signalling, #9677 matched with Alexa Fluor^®^ 594 (Invitrogen, A-11012).

### Statistical analysis

Data are presented as mean ± standard error of the mean (SEM). One-way ANOVA with Tukey post-correction was used to test for significance (P < 0.05).

## Results and discussion

**Fig. 2:**
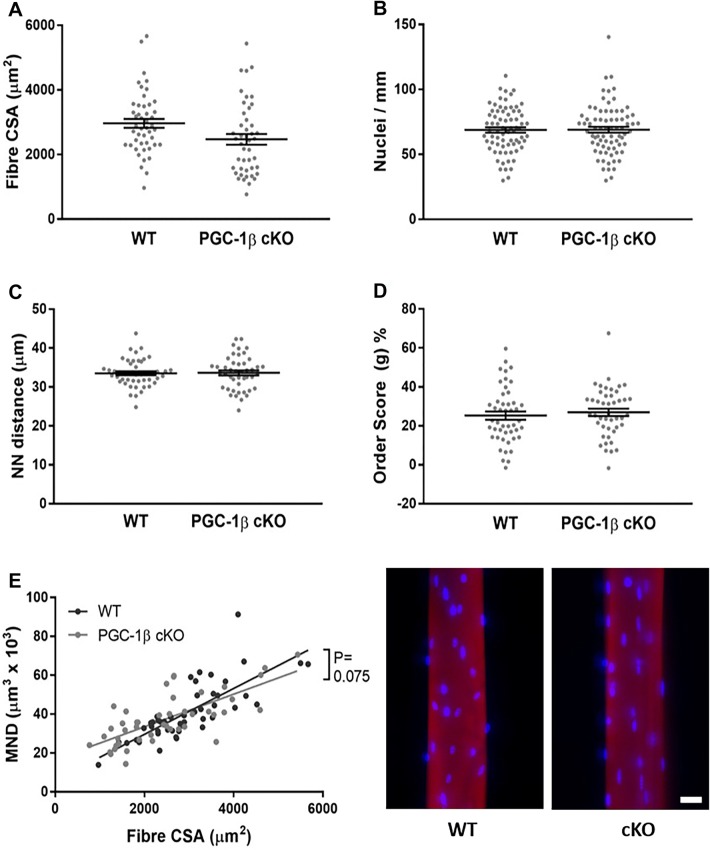
4 weeks of PGC1-β ablation did not result in differences in nuclear distribution. Single myofibres were isolated from cKO (PGC1-β muscle-specific knockout) and WT (wild-type) rodents. These were then stained for actin (rhodamine-conjugated phalloidin, Molecular Probes, R415, red) and nuclei (DAPI, blue). WT and cKO fibres were imaged using a confocal microscope, representative images are shown. Scale bar: 25 µm. **a**–**e** Data are presented as mean ± SEM, and as scatterplots where individual points correspond to single muscle fibres. (Color figure online)

**Fig. 3:**
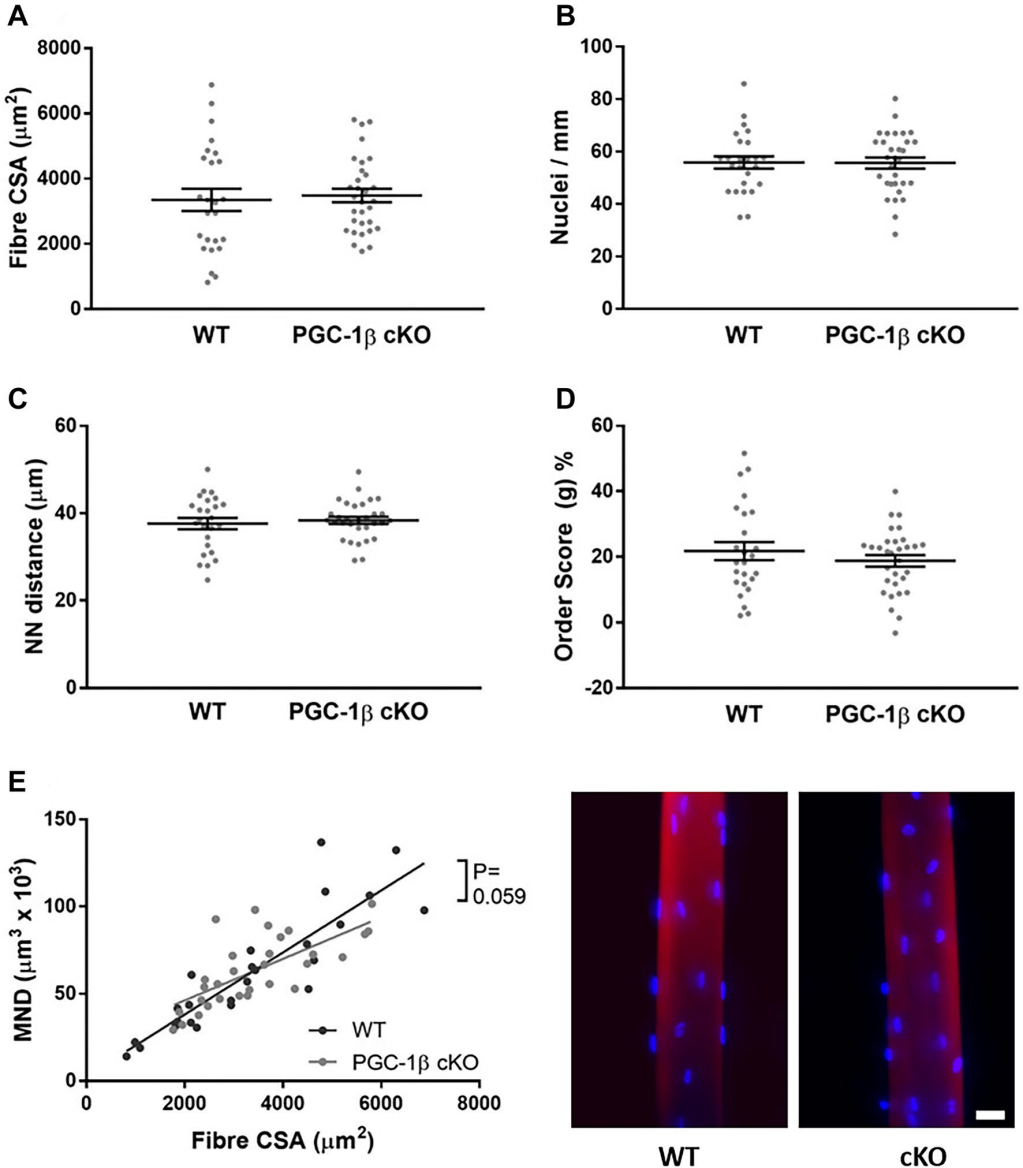
After 10 weeks of PGC1-β ablation, nuclear organisation is not altered. As for Fig. 1, muscle fibres were isolated from cKO (PGC1-β muscle-specific knockout) and WT (wild-type) rodents. These were then stained for actin (rhodamine-conjugated phalloidin, Molecular Probes, R415, red) and nuclei (DAPI, blue). WT and cKO fibres were imaged using a confocal microscope, representative images are shown. Scale bar: 25 µm. **a**–**e** Data are presented as mean ± SEM, and as scatterplots where individual points correspond to single muscle fibres. (Color figure online)

To investigate whether PGC1-β modulates nuclear distribution, single muscle fibres from PGC-1β^(i)skm-/-^ (PGC-1β cKO) mice, and control (WT) littermates were isolated 4 and 10 weeks after gene ablation, stained and imaged (Figs. 2, 3).

Surprisingly, after 4 and 10 weeks of PGC1-β ablation, no significant differences in fibre cross-sectional area (CSA), nuclei number per mm length, nearest neighbour distance (NN), order score and MND size were observed between WT and PGC-1β cKO mice (Figs. 2, 3). Hence, in contrast to PGC1-α (Ross et al. 2017), our data suggest that PGC1-β does not play essential role in nuclear organisation or MND determination. Note that our analysis here was run on muscle fibres extracted from tibialis anterior muscles mainly expressing IIx and IIb myosin heavy chain isoforms. Such expression was also present in the EDL muscles we used in our previous study where we observed differences with varying levels of PGC1-α (Ross et al. 2017).

**Fig. 4:**
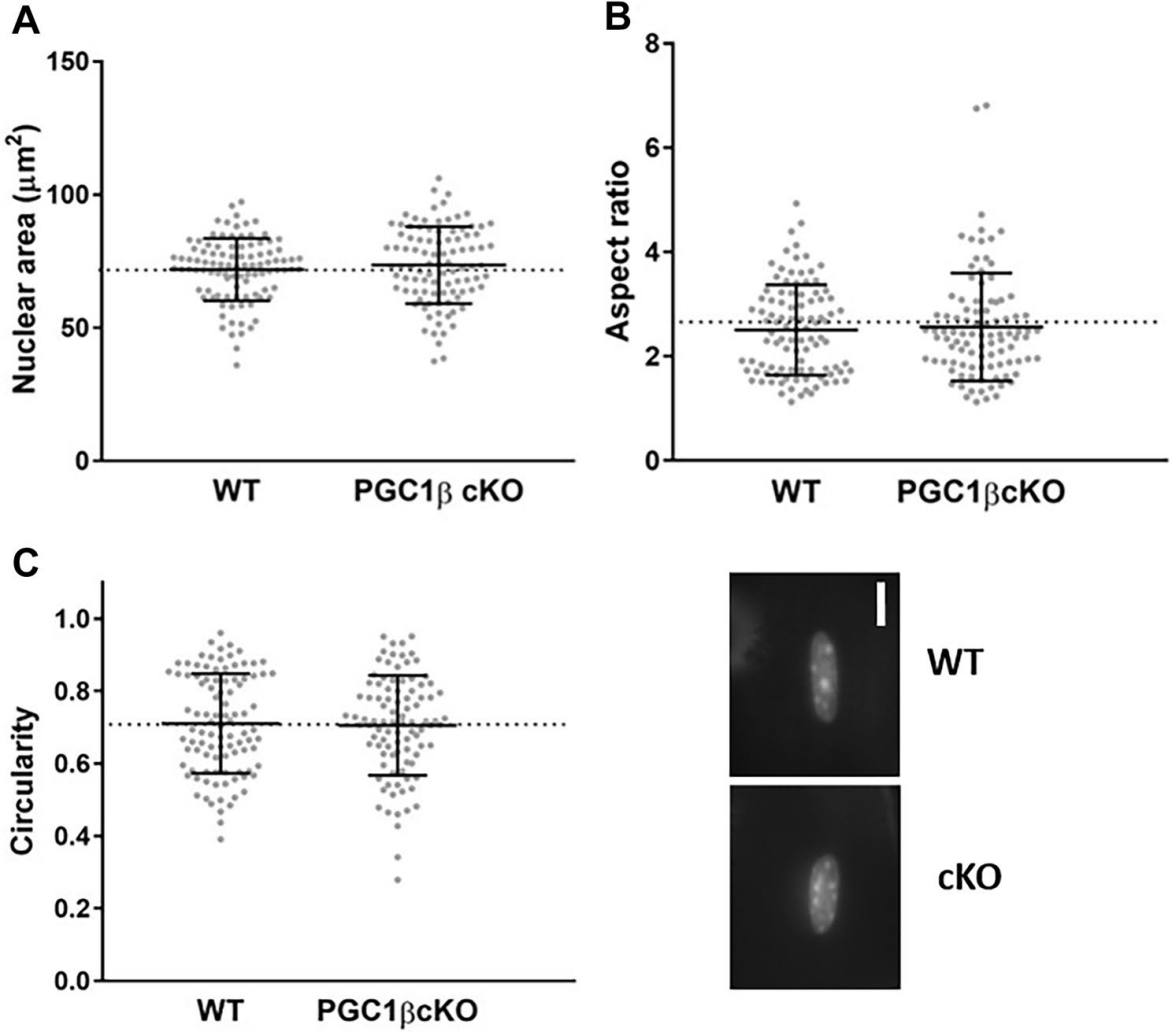
4 weeks of PGC1-β ablation had no effect on nuclear morphology. Typical nuclei from cKO (PGC1-β muscle-specific knockout) and WT (wild-type) rodents are presented. These were stained using DAPI. Scale bar is 5 µm. **a**–**c** Data are presented as mean ± SEM, and as scatterplots where individual points correspond to single muscle fibres

**Fig. 5:**
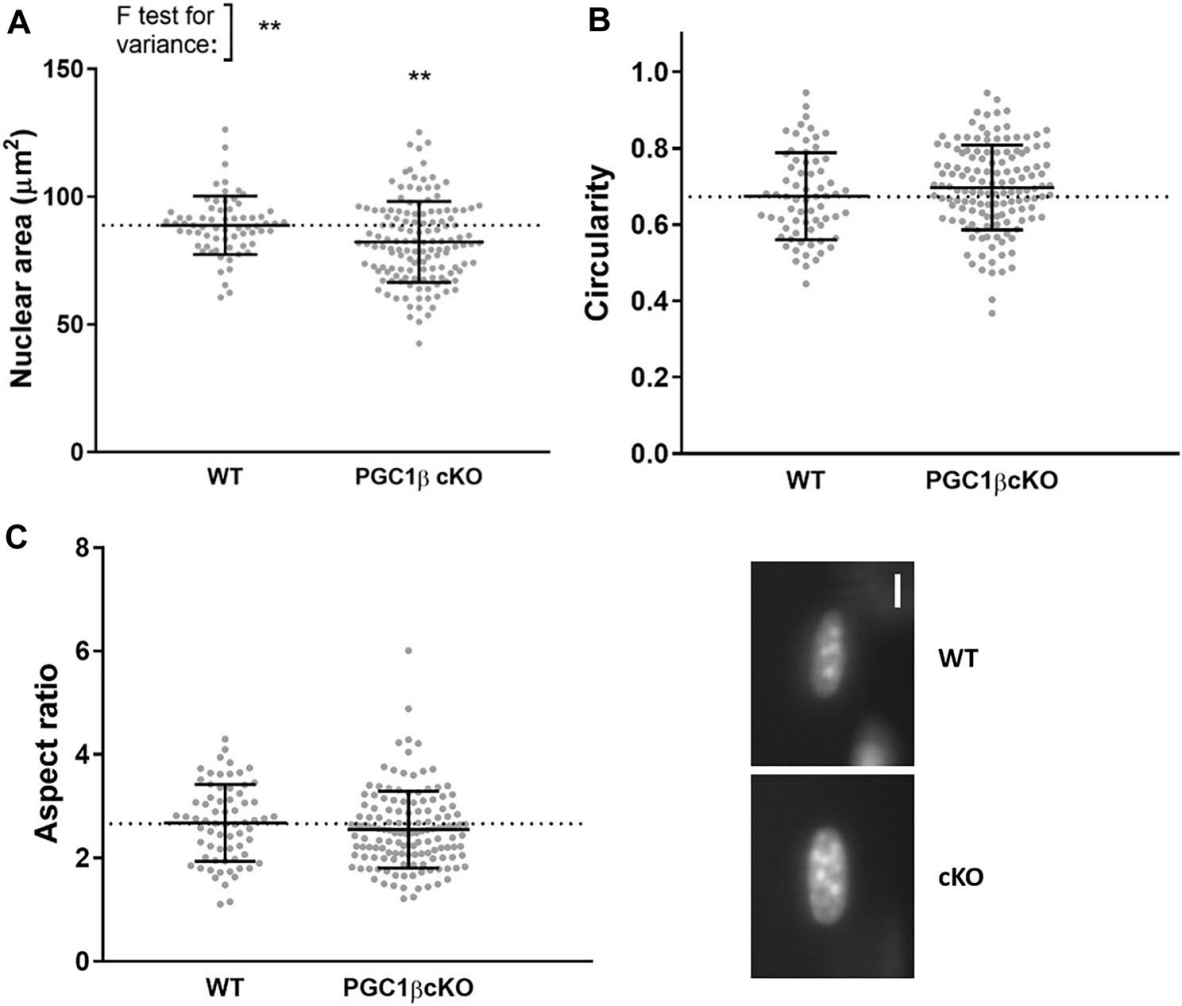
After 10 weeks of PGC1-β ablation, nuclear shape is changed. Typical nuclei from cKO (PGC1-β muscle-specific knockout) and WT (wild-type) rodents are presented. These were stained using DAPI. Scale bar is 5 µm. **a**–**c** Data are presented as mean ± SEM, and as scatterplots where individual points correspond to single muscle fibres

In addition to assessing nuclear distribution, since nuclear shape is related to transcriptional control and given the role of PGC1-β as a transcription factor, we also evaluated a number of morphological parameters at physiological sarcomere length (Figs. 4, 5). Four weeks after PGC1-β ablation, no significant differences in nuclear area, aspect ratio and circularity were noticed between WT and PGC-1β cKO mice (Fig. 4). Nevertheless, after 10 weeks of PGC1-β ablation, nuclei from PGC-1β cKO mice were smaller when compared with WT rodents (Fig. 5). Hence, the ablation does not result in an immediate effect on nuclear morphology but a gradual and/or delayed subtle change (approximately 10% difference between groups). This indicates that PGC1-β plays a minor role in determining nuclear shape.

**Fig. 6:**
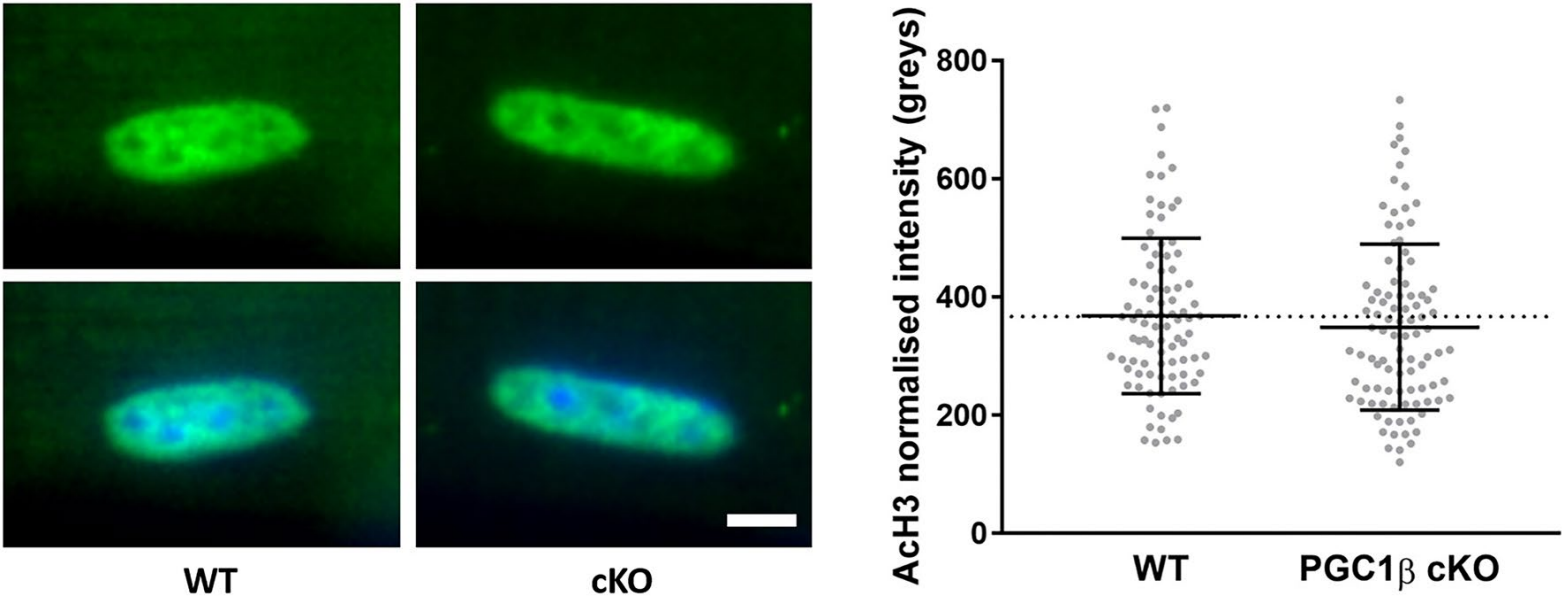
10 weeks of PGC1-β ablation had no effect on AcH3 intensity. Single myofibres were isolated from cKO (PGC1β muscle-specific knockout) and WT (wild-type rodents). These were then stained for nuclei (DAPI, blue) and Acetyl-histone H3 (rabbit anti-acetylhistone H3, Cell Signalling #9677, green) as a measure of global transcriptional activity in each nucleus. WT and cKO fibres were then imaged using a confocal microscope. Representative images of individual nuclei are shown. The top two WT and cKO images show just the acetyl-histone H3 stain. There is no significant difference in nuclear fluorescence intensity. The bottom WT and cKO images show a merge of both the DAPI and acetyl-histone H3 stains. Scale bar: 5 µm. Data are then presented as mean ± SEM, and as scatterplots where individual points correspond to single nuclei. (Color figure online)

As mentioned above, because of the late onset of such change, our findings highlight an indirect PGC1-β effect on the regulation of nuclear shape. To further investigate whether the change in nuclear morphology is associated with modifications in global transcriptional activity, we measured acetyl-histone H3 (AcH3) fluorescence intensity. We did not observe any significant difference in the AcH3 fluorescence intensity between WT and PGC-1β cKO mice (Fig. 6). The AcH3 staining is just a measure of global transcriptional activity. It is therefore plausible that other unidentified alterations of specific subsets of transcriptional pathways occur.

To conclude, the aim of the current manuscript was to define whether PCG1-β plays a role in modulating nuclear distribution and shape. For that, we used PCG1-β cKO mice. Unlike our initial hypothesis, our results suggest that PGC1-β does not directly modulate MND size or overall nuclear organisation within individual myofibres. On the other hand, some minor differences in nuclear shape were observed. PGC1-β and PGC1-α are homologues, even though they are both important for the regulation of OxPhos gene expression and mitochondrial biogenesis, they have distinctive roles within skeletal muscle (since PGC1-α ablation or overexpression resulted in modulation of MND size). PGC1-α which is the predominant isoform may be the driver of skeletal muscle metabolism and function whereas PGC1-β may only be one of the modulators. This would have to be tested in a further study.
